# Round the Hospitals

**Published:** 1918-11-09

**Authors:** 


### ROUND THE HOSPITALS.

THE luncheon party given at the Trocadero on October 30 by representative women in honour of the Matrons-in-Chief of the Military Nursing Services of the Crown was a notable affair. The guests of honour were Dame Ethel Becher, Q.A.I.M.N.S.; Dame Maud McCarthy (France); Miss Sidney Browne, T.F.N.S.; Miss McDonald, Canadian Nursing Service; Miss Thurston, New Zealand Nursing Service. Miss Conyers, Australian Nursing Service, was prevented by illness from being present, as was also the representative of the South African Nursing Service. Princess Louise presided. The King sent roses from Windsor Castle. Queen Alexandra sent grapes from Sandringham. A telegram in appreciation of the Queen's constant solicitude for the wounded and encouragement of nursing effort was dispatched during lunch, and Her Majesty sent a cordial response testifying to her full appreciation of the splendid work achieved by nurses throughout the Empire.

IT is always a delicate and dangerous task to tell people to their face how greatly they are admired, and to receive such tributes gracefully is even more perplexing. There could not be two opinions, however, about the sincerity and warmth of Princess Louise's words to the women whose "tact, knowledge, patience, sympathy, true womanliness, and fearless, untiring devotion" had directed the nursing Services during the war. Dame Ethel Becher accepted the praises lavished on the nursing profession of Great Britain and the Dominions by the Princess and by Mrs. Humphry Ward with a dignity not lacking a touch of heartfelt rejoicing over the achievements of the nurses. She gave some interesting figures. On August 1, 1914, the regular service of Army nurses consisted of 290 members and the reserve of 173, a total of 463. On mobilisation, by the help of the civil hospitals, they were able at once to call upon 800 trained nurses for duty with the units of the original Expeditionary Force. They were supplied without a hitch; each nurse arrived at her post and there was not one absentee. At the present time the number of nurses in Q.A.I.M.N.S., and reserve is approximately 8,000, and there are some 6,000 V.A.D. nursing members. Miss Becher, in conclusion, expressed her personal gratitude to the Matrons-in-Chief of the Dominions for the help and co-operation received from them.

AT the Investiture held at Buckingham Palace on November 1 the following were admitted Associates of the Royal Red Cross:—Matron Emmeline Wilding, T.F.N.S.; Sister Hilda Macdonald, Canadian Army Nursing Service; Miss Dalsie Buffard and Miss Lucy Purcell, V.A.D.

MISS E. F. TICE has been appointed matron of Charing Cross Hospital in succession to Miss Heather-Bigg, whose resignation we announced in our issue of Oct. 12. Miss Tice, who was trained at St. Bartholomew's Hospital, has been matron of the Samaritan Free Hospital since 1905, and previous to this appointment she was successively night superintendent, ward sister, and assistant-matron of University College Hospital. Miss Tice's experience of nursing administration in metropolitan hospitals is thus seen to have been long and varied. The responsibilities of the matron to important hospitals with training-schools tend to increase continually.

IN a note on the 26th ultimo we called attention to the zeal of many matrons who make a practice of providing their institutions as far as possible with home-made jam and other food accessories. This week we have the pleasure to report the receipt from a matron to a fever hospital in Banffshire, where they grow all their own vegetables with the help of one man only, and where the matron has made all the jam and marmalade used in this hospital, of samples of jams, jellies, other products, and vegetables. For the last two years, owing to a great shortage of fruit, and the absence of oranges, only rhubarb has been available for the matron's use, a large quantity of which she has turned into jam. Neighbours sent her some sweet green gooseberries, out of which she made jam, and by careful testing she was able to save sugar by not using the skins with the fruit. She coloured the product with cochineal and added sago to thicken it. Another plan pursued was to convert a present of apples into apple jelly and apple butter, the latter being again coloured with cochineal and thickened with sago. We have received samples of all these delicacies, which do credit to the matron and are grateful to the human palate.

IN thanking Miss E. Law for all the trouble she has taken and congratulating her on the excellence of the results she has secured for her hospital, we are happy to report that a skilled and knowledgeable judge of garden produce reports that three of the four samples of potatoes—namely, British Queens, Arran Chiefs, and Golden Wonders—are fine specimens, that the cabbages and savoy cabbage are splendid, and everything demonstrates the excellence of the service rendered to the Campbell Hospital by those who have produced these vegetables under the practised eye of the matron Another sample of potatoes, Champions, is good, for it illustrates the comparative unpopularity of this variety on account of the depth, size, and number of eyes, which involve waste. A fair sample of beetroot is disappointing. Next year's yield would be greatly increased in value if the ground is trenched in the early autumn. The shallots are the best we have seen this year, and are both attractive and finely grown. Comparisons are odious, but we may add that the carrots sent are shapely and most excellent, this variety of short carrot being the best to be met with in the London market this season. In thanking Miss E. Law and congratulating her on the successful

**ROUND THE HOSPITALS—(*continued*).**

results achieved under her management we have confidence the progress made must stimulate other matrons to go and do likewise. Other results will be welcome.

AN Irish correspondent draws our attention this week to the fact that £10,000 has been collected as a part of the Nation's Tribute to Nurses. The money was collected in Ireland, and in Ireland it is to be distributed. So far as we are aware it has never been definitely decided whether or not it is to form a part of the Nation's Tribute, although it was inaugurated by the President. When Sir Arthur Stanley addressed a large and influential public meeting in Dublin he waived every claim except this, but he pressed home the request with all his force that whatever sum was collected it should form a part of the national and imperial movement. No man could possibly have taken a broader or more liberal view of the situation. And no man or woman could have achieved such success in the collection of the Fund as Lady Arnott. Wholeheartedly she threw herself into it, first by handsomely subscribing herself, secondly by propaganda through the *Irish Times*, of which Sir John Arnott is the Chairman, and, thirdly, by propaganda amongst her friends all over Ireland. The Nurses' Tribute Fund in Ireland owes its inception and its success to Sir Arthur Stanley and Lady Arnott; of this there is no room for doubt. The question arises, who is to administer the Fund?

THE College of Nursing stands for the unity and betterment of British Nurses. Opponents of the College of Nursing stand for faction and disunion. These are broad principles, and the time has come when a definite stand must be taken. The administration of the Irish Tribute must not be handed over to the Bolshevists, to the people who run with the hare and bark with the hounds. Those who collect the Funds must dictate their administration, and if Lady Arnott and Sir Arthur Stanley have made an Irish Tribute Fund for Ireland, it is clearly their office, and none other's to nominate the Commissioners who are to be responsible for its distribution.

INFLUENZA has been taking heavy toll from the nursing profession. We regret to record the death from influenza and pneumonia on October 26, after a few days' illness, of Miss Adelaide Beatrice Aked. She was trained at the Hull Royal Infirmary, where she afterwards became sister of the Women's Surgical Ward. An impressive Memorial Service was held in the hospital chapel, which was attended by representatives of the Board of Management, the honorary medical and resident and nursing staff, as well as relatives and friends of the deceased, to show the respect in which Miss Aked was held. Her devoted and conscientious work will be greatly missed. In Lincoln the death of Nurse Muriel (Sister Vousden), after five day's illness, has caused widespread grief. For sixteen years she was attached to the Ancient Order of Foresters, latterly for some years as Chief Ranger of the Court Henrietta Bromhead, and exercised the duties of district nurse and sick visitor, "carrying the watchword of the Order, 'Sympathy,' to every house she visited." Her funeral was very impressive from the number of mourners present who had been her patients.

IT is with much regret that we record the death of Miss H. M. Cottingham, who was acting matron of the Inns of Court O.T.C. Detention Hospital, Berkhamsted. She contracted influenza on Saturday, October 19, which was followed by pneumonia. She became rapidly worse and died the following Saturday night, October 26, in spite of all the skill and care lavished upon her. As Miss Cottingham's home is in Ireland she was buried at Berkhamsted with military honours on October 29. The authorities of the Druids' Red Cross Hospital, Wavertree, Liverpool, have just reported the death of Sister E. J. Hewitt on October 29 from pneumonia following influenza. She was devoted to her work, and was held in high esteem by those with whom she worked.

A WARM tribute was paid to the work of the V.A.D.s by Colonel J. A. Hughes, C.B., C.B.E., County Director for Glamorgan, at a meeting of the Glamorgan Territorial Force Association at Cardiff on October 24. He referred with pride to the fact that the county ranked seventh in the kingdom in this matter, and remarked, amid applause, that, in view of the strong competition of other organisations, it was gratifying to find so large a proportion of their 2,850 women V.A.D.s were sticking to their work without pay. The number of auxiliary hospital beds in Glamorgan has increased from 2,424 to 3,043 in the past twelve months, and it is hoped to meet the pressure still existing by the early addition of 300 to 400 extra beds.

THE annual meeting of the Ladies' Association of the Great Northern Central Hospital was held on October 24, when some of the members' work accomplished during the year was on view. There was a large attendance, and the matron, Miss A. M. Bird, R.R.C., spoke warmly of the help accorded to her by the Association. In many respects these Ladies' Hospital Associations were pioneers, and in the development of centres for war depôts their experience was very valuable. It is probable that after the war they will enlarge their scope, and that surgical dressings and other necessary articles of hospital equipment will be to a great extent provided in the future by voluntary workers.

THE Liverpool Centre of the College of Nursing has reaped the sum of £578 3s. in aid of the Nation's Fund for Nurses. No less than £128 of this sum has been collected at a drawing-room meeting held at the house of Dr. and Mrs. Bickerton. The Lord Mayor, who presided on this occasion, showed himself an enthusiastic supporter of the movement for placing the nursing profession on

**ROUND THE HOSPITALS—(*continued*).**

an assured basis. He was followed by Mrs. Martin Harvey, who recited the "Hymn of Love," which has been composed as an answer to Germany's "Hymn of Hate." The West Derby Union officers raised £173 15s. 10d. for the Fund, of which £100 was assigned to the Nation's Fund for Nurses Benevolent Fund Branch, and £71 to the Liverpool Branch of the College. Hull has sent £86 this year to the Nation's Fund, the result of an "At Home" given by the Lady Mayoress at the Guildhall—when Mr. Martin Harvey gave a recitation, and Mrs. Martin Harvey made a moving appeal for the Fund. The committee of the Hull Branch hope to raise a further sum of money in the spring of next year.

NURSES' PRIZE DAY is always celebrated with great ceremony at the East Suffolk and Ipswich Hospital, and the proceedings last month were unusually full of interest, and were attended by a large gathering. The chairman laid stress on the difficulties of the past year and eulogised the services of the matron, Miss Deane, and of Sister Collet. Prizes of books were bestowed on the nurses who stood first and second in their respective divisions, and the medals won in accordance with the results of two examinations conducted by Mr. Russell Howard, M.S., F.R.C.P., were conferred as follows:—*Gold Medal*, Nurse Saunders; *Silver Medal*, Nurse Sinclair; *Bronze Medals*, Nurses Shephard, Bingham, Scarfe, Cracknell, Sayer, Buck, Denny, Singleton, Francis, Wright, Nunn, and Parr. Miss Deane gave a telling address, adjudging her own prize, a travelling clock, to Miss Cicely Amy Buck.

THE committee of the Royal Victoria and West Hants Hospital, Bournemouth, is raising salaries as follows:—Ward sisters at present in receipt of £50 a year will now have £55 in their second year, and £60 in their third year. Departmental sisters are to receive "various additions" besides. All sisters are to have an allowance of £3 for uniform. Staff nurses are to have £40, £45, with £2 10s. for uniform; probationers, £15, £20, £28, with uniform allowance of £3 10s. for the first year and £3 after. The probationers have been receiving £10, £15, £20. We do not quite understand why staff nurses receive 10s. less for uniform than either probationers or sisters. In any case the uniform allowance is so low as seriously to destroy the effect of the increase in salaries.

OUR readers have followed the fortunes of the Elsie Inglis Transport attached to the Scottish Women's Hospital Unit in their tragic retreat, and will be interested to learn that it has been reconstituted and is now swiftly following the line of the victorious armies on their march through the recovered tracts of Serbia. The Elsie Inglis travelling hospital and ambulance did fine work at the commencement of the advance along mountain tracks bordered by precipices, and their services were sorely needed. The wounded were brought over the passes lying on straw in rough and jolting carts through the devastated country, and the cars provided by the Transport to meet the wounded and convey the worst cases down to the hospital undoubtedly saved many lives.

THE letter of an M.A.B. nurse published in the Editor's Letter-Box in our issue of October 19 calls attention to the extreme dislike to being "warded" felt by all nurses alike. It may seem a little thing, but it is always the little things that count in life. To succumb to infection is not always or necessarily a thing to be dreaded. It may mean merely a few weeks' inaction under very favourable circumstances, with subsequent immunity. But to be cast down from the status of a nurse to that of a patient in one of the ordinary wards is very bitter to some, and the ordeal, of course, is worse in contemplation than in reality. The provision of a comfortable sick-room bay for the nurses in all hospitals for infectious diseases is essential to the well-being of the staff. It might remove a stumbling-block from the path of many good candidates.

THE annual meeting of the Poor-Law Infirmary Matrons' Association on the 26th ultimo was made the occasion for the presentation of a handsome purple leather hand-bag mounted in silver-gilt to Miss Stansfeld, on her retirement from service under the Local Government Board for twenty-five years. An old silver chatelaine and a blotter have since been presented. Miss Barton, in making the presentation on behalf of the Association, dwelt on Miss Stansfeld's ready help and sympathy, which she extended freely to all the members of the Association. Miss Stansfeld, who has had considerable opportunity to judge the work of infirmary matrons, in her reply spoke appreciatively of what they had done and of the pleasure her association with them had given her. At the same meeting of the Association the names of the honorary officers and committee who have been elected for service during the forthcoming year were announced. They are: Miss Barton, R.R.C. (Matron Chelsea Infirmary), President; Miss Alsop (Matron Kensington Infirmary), Hon. Sec.; Miss Inglis (Matron Shoreditch Infirmary), Hon Treasurer; the committee consisting of Miss Bodley (Selly Oak), Miss Clark (West Ham), Miss Dodds, R.R.C. (Bethnal Green), Miss Dowbiggin, R.R.C. (Edmonton), Miss Hannaford, O.B.E. (Poplar and Stepney Sick Asylum), Miss Masters (Leicester), Miss Mowatt (Whitechapel), Miss Myles (Brighton), Miss Elna Smith (City of Westminster, Hendon), Miss M. Smith, R.R.C. (West Didsbury, Manchester), Miss Williams (Cardiff), Miss Seymour Yapp (Ashton under Lyne).

**☞ IT is our invariable practice to pay for all items of news which we may publish. The minimum payment is 5s. Paragraphs of about twenty lines in length—that is to say, of 150 words or less—are preferred. Contributions should be addressed to The Editor, THE HOSPITAL, 28 and 29 Southampton Street, Strand, London, W.C. 2, and, to be in time for the current week's issue, should reach him by the first post on Mondays.**

